# Higher expression of monocyte chemotactic protein 1 in mild COVID-19 patients might be correlated with inhibition of Type I IFN signaling

**DOI:** 10.1186/s12985-020-01478-9

**Published:** 2021-01-07

**Authors:** Xueyan Xi, Yang Guo, Min Zhu, Yuhui Wei, Gang Li, Boyu Du, Yunfu Wang

**Affiliations:** 1 Institute of Basic Medical Science, Hubei University of Medicine, Shiyan, No. 30 Renmin Nanlu, Shiyan City, Hubei Province 442000 People’s Republic of China; 2 Hubei Key Laboratory of Embryonic Stem Cell Research, Hubei University of Medicine, Shiyan City, People’s Republic of China; 3 Institute of Neuroscience, Hubei University of Medicine, Shiyan City, People’s Republic of China; 4 Renmin Hospital, Hubei University of Medicine, Shiyan City, People’s Republic of China

**Keywords:** COVID-19, Chemokines, MCP-1, IFN signaling, Mild

## Abstract

**Background:**

Chemokine levels in severe coronavirus disease 2019 (COVID-19) patients have been shown to be markedly elevated. But the role of chemokines in mild COVID-19 has not yet been established. According to the epidemiological statistics, most of the COVID-19 cases in Shiyan City, China, have been mild. The purpose of this study was to evaluate the level of chemokines in mild COVID-19 patients and explore the correlation between chemokines and host immune response.

**Methods:**

In this study, we used an enzyme-linked immunosorbent assay to detect serum levels of chemokines in COVID-19 patients in Shiyan City. Expression of chemokine receptors and of other signaling molecules was measured by real-time polymerase chain reaction.

**Results:**

We first demonstrated that COVID-19 patients, both sever and mild cases, are characterized by higher level of chemokines. Specifically, monocyte chemotactic protein 1 (MCP-1) is expressed at higher levels both in severe and mild cases of COVID-19. The receptor of MCP-1, C-C chemokine receptor type 2, was expressed at higher levels in mild COVID-19 patients. Finally, we observed a significant negative correlation between expression levels of interferon (IFN) regulatory factor 3 (IRF3) and serum levels of MCP-1 in mild COVID-19 patients.

**Conclusion:**

Higher expression of MCP-1 in mild COVID-19 patients might be correlated with inhibition of IFN signaling. The finding adds to our understanding of the immunopathological mechanisms of severe acute respiratory syndrome coronavirus 2 infection and provides potential therapeutic targets and strategies.

## Background

An outbreak of coronavirus disease 2019 (COVID-19) caused by severe acute respiratory syndrome coronavirus 2 (SARS-CoV-2) has rapidly spread throughout the world [1, 2]. Globally, as of December 2020, there have been 65,870,030 confirmed cases of COVID-19, resulting in 1,523,583 deaths (https://covid19.who.int/). According to epidemiological statistics, most COVID-19 cases (about 80%) are classified as mild [3, 4]. Most COVID-19 patients in Shiyan city, China, had mild cases [4]. For this reason, it is necessary to study the immunological characteristics of patients with mild COVID-19 and define a suitable therapeutic strategy for these cases.

Immune response is the body’s defense mechanism against viral infection, involving the innate and adaptive immune responses. However, excessive immune responses after infection, also called a cytokine storm, have been found to be associated with extreme levels of pro-inflammatory cytokines. Patients infected with SARS-CoV-2 show higher leukocyte counts, abnormal respiratory findings, and increased plasma levels of pro-inflammatory cytokines. The direct cause of death from acute COVID-19 might be the damage to lungs and many other organs, resulting in multi-organ failure, caused by cytokine storm [5].

Preliminary studies have shown that SARS-CoV-2 infection can trigger a cytokine storm, increasing the levels of a variety of cytokines, including chemokines [5–7]. Chemokines are low-molecular-weight proteins with powerful chemoattractant activity that play roles in the immune cell recruitment during inflammation; they are classified by their chemical structure into the C, CC CXC and CX3C families [8]. Their chemoattractant ability is caused by their binding to their receptors. Chemokine receptors are G-protein-coupled receptors that span seven transmembrane and are expressed on leukocytes and endothelial cells, etc. [9]. Serum chemokine levels are elevated in patients with COVID-19, and they are even higher in those who required intensive-care unit (ICU) admission, suggesting a relationship between chemokines and both lung damage and disease severity [10]. Although chemokine concentrations are also elevated in mildly ill COVID-19 patients [6], it is not clear whether high expression of chemokines could be used as a marker for the diagnosis and prognosis in mild COVID-19.

Type I interferons (IFNs), including IFN-α and IFN-β, have broad-spectrum antiviral activities: they act by inducing an antiviral response and mediating innate and adaptive immune responses [11]. Viral infection of cells activates several cellular transcription factors, such as IFN regulatory factor 3/7 (IRF3/7) and NF-κB, which in turn activate the expression of a number of interferon-stimulated genes (ISGs) and exert antiviral effects [12, 13]. The activation of transcription factors also induces that the secretion of chemokines, which further recruit and coordinate specific subsets of leukocytes such as neutrophils and monocytes [14, 15]. This means that chemokines secretion might be related to the release of IFN, but the specific relationship between chemokines and IFN in the process of SARS-CoV-2 infection has not been clearly established.

Clinically, Type I IFNs are already approved for use in the treatment of certain cancers, autoimmune disorders, and viral infections [16]. As they are currently in clinical trials evaluating their ability to treat Middle East respiratory syndrome coronavirus (MERS-CoV) [17], they have been proposed for the treatment of COVID-19, but there is currently no evidence from laboratory testing against SARS-CoV-2.

In this study, we detected serum concentrations of chemokines and expression levels of chemokine receptors in peripheral-blood mononuclear cells (PBMCs) from COVID-19 patients in Shiyan City in order to evaluate the role of chemokines in mild COVID-19. Then, we evaluated the level of IFN-β and the relationship between *IRF3* and MCP-1 in order to prove the correlation between MCP-1 and IFN signaling. Our results suggest that higher expression of monocyte chemotactic protein 1 (MCP-1) in mild COVID-19 patients might be correlated with inhibition of IFN signaling.

## Methods

### Subjects

We recruited 10 severely and 30 mildly ill COVID-19 patients from Xiyuan Hospital and Renmin Hospital, Shiyan City. Severity of COVID-19 symptoms was graded according to China’s *National Health Commission Guidelines for Diagnosis and Treatment of SARS-CoV-2 Infection* (7th ed. http://www.nhc.gov.cn/yzygj/s7652m/202003/a31191442e29474b98bfed5579d5af95.shtml). Our study protocol received approval from the Clinical Ethics Committee of Hubei University of Medicine, Shiyan City (No. 2020-TH-017). Ten healthy subjects, all of whom were free from tumors, infections, and other diseases, were recruited as a control group. All individuals gave their informed consent to participate. Basic information of COVID-19 patients and healthy controls is listed in Table 1.

**Table 1:** Primer sequences of the chemokine receptors and *IRF3*

Features	Severe COVID-19	Mild COVID-19	Healthy controls
Mean age (year)	56.3	44.6	42
Gender (female/male)	30%	40%	40%
Average time of hospital (day)	28.4	23.4	–
Diabetes	20%	16.7%	–
Heart disease	20%	20%	–
Hypertension	40%	23.3%	–
Cancer	0%	0%	–

According to China National Health Commission Guidelines for Diagnosis and Treatment of SARS-CoV-2 infections (7th, ed.) issued by 2020

### Isolation of peripheral-blood mononuclear cells (PBMCs)

PBMCs were isolated from peripheral-blood samples of study participants via density gradient centrifugation using Ficoll-Paque media (Sigma-Aldrich, No. 17144002). We isolated 1 × 10^6^ PBMCs/mL peripheral blood. Total ribonucleic acid (RNA) was extracted from 3 × 10^6^ isolated PBMCs using TRIzol reagent (Thermo Fisher Scientific, No. 15596026) per manufacturer’s protocol.

### Real-time PCR

After isolating total RNA from PBMCs, we reverse-transcribed it into complementary deoxyribonucleic acid (cDNA). Real-time quantitative polymerase chain reaction (RT-qPCR) using SYBR Premix Ex Taq (TaKaRa, No. RR820A) was performed to quantify chemokine receptors, transcription factors, and glyceraldehyde 3-phosphate dehydrogenase (GAPDH) levels. Each reaction tube contained 20 μL reaction mixture, including 10 μL SYBR Premix Ex Taq, 1 μL of each 10-μM primer, 1 μL cDNA template, and 7 μL double-distilled water (ddH_2_O). The program was as follows: denaturation at 95° C for 10 min, followed by 40 cycles of 15 s denaturation at 95 °C and 60 s annealing/extension at 56 °C. We used the comparative Ct method to calculate the relative abundance of different genes compared with expression of GAPDH. Primer sequences are listed in Table 2.

**Table 2:** The primer sequence

	Gene name	Primer sequence
1	*CCR2*	Up: 5′-AAGAGGCATAGGGCAGTGAG-3′
		Down: 5′-GGGATTGATGCAGCAGTGAG-3′
2	*CXCR2*	Up: 5′-GCATCAGTGTGGACCGTTAC-3′
		Down: 5′-GGCTGGGCTAACATTGGATG-3′
3	*CXCR3*	Up: 5′-TGGTGGACATCCTCATGGAC-3′
		Down: 5′-CAAGAGCAGCATCCACATCC-3′
4	*IRF3*	Up: 5′-GACCCTCACGACCCACATAA-3′
		Down: 5′-CAGAAGTACTGCCTCCACCA-3′

Primer sequences of the chemokine receptors and *IRF3*

### Enzyme-linked immunosorbent assay (ELISA)

We determined concentrations of chemokines, MCP-1, interferon-inducible protein-10 (IP-10), interleukin-8 (IL-8), and IFN-β in the serum of COVID-19 patients and healthy controls using ELISA kits (Shenzhen Dakewei Bio-Engineering Co., Ltd. Nos. 1117392, 1110802, and 1117452; and Sanying Precision Instruments Co., Ltd. No. KE00187) per manufacturers’ instructions. Undiluted serum samples were added to the pre-coated ELISA plate and incubated for 2 h, after which enzyme-labeled antibody was added. After washing the plate five times, we added the substrate for color development and then determined absorbance using an Epoch microplate spectrophotometer (BioTek Instruments, Inc.) at 490 nm.

### Statistical analysis

We used analysis of variance (ANOVA) tests to compare plasma chemokine levels among the COVID-19 and healthy controls. Spearman’s correlation coefficient (SCC) was used for linear-correlation analysis between expression levels of plasma chemokines and *IRF3*. We analyzed all data using SPSS version 19.0 and GraphPad software version 5.0. *P* < 0.05 was considered statistically significant.

## Results

### MCP-1 levels in mild COVID-19 patients are higher than in healthy controls

**Fig. 1:**
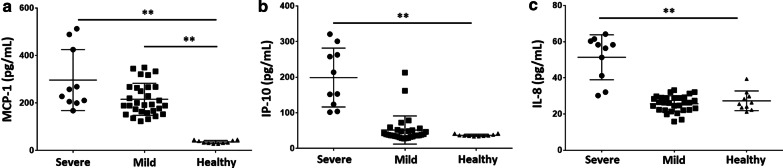
MCP-1 levels in mild COVID-19 patients are higher than in healthy controls. Chemokine levels in serum of study subjects were detected by ELISA. **a** MCP-1 levels in serum of peripheral blood from COVID-19 patients (n = 40) were higher than those in serum from healthy controls (n = 10). **b** IP-10 and **c** IL-8 levels in serum of peripheral blood from severe COVID-19 patients (n = 10) were higher than those in serum from mild COVID-19 patients (n = 30) and healthy controls (n = 10). **P < 0.01

The basic information of severely and mildly ill COVID-19 patients listed in Table 1 suggests that our statistical analysis found no remarkable differences between them in length of hospital stay or certain complications. To further clarify the different role of chemokines in severe and mild COVID-19 patients, we determined expression levels of three chemokines- MCP-1, IP-10 and IL-8- in the serum among either severe, mild COVID-19 patients, or healthy controls. MCP-1 upregulation was observed in most COVID-19 patients, whether cases were severe (296.7 pg/mL ± 128 pg/mL) or mild (215.9 pg/mL ± 67.4 pg/mL), versus healthy controls (36.2 pg/mL ± 6.7 pg/mL; Fig. 1a; *P* < 0.01). IP-10 was upregulated in severe COVID-19 patients (199.2 pg/mL ± 82.6 pg/mL), but we found no difference between mild patients (51.7 pg/mL ± 39.4 pg/mL) and healthy controls (37.1 pg/mL ± 3.1 pg/mL) (Fig. 1b).

Meanwhile, severe COVID-19 patients also showed upregulation of IL-8 (51.3 pg/mL ± 12.4 pg/mL vs 27.2 pg/mL ± 5.4 pg/mL in healthy controls; Fig. 1c). Recent reports indicate that MCP-1, IP-10, and IL-8 levels are higher in COVID-19 patients and even higher among those admitted to ICU [10]. We also found that the expression levels of the three chemokines were increased in severe COVID-19 patients. It is suggested that chemokines play an important role in patients with severe COVID-19; the upregulation of MCP-1 in mild COVID-19 suggested that it might also play a role in pathogenesis thereof.

### CCR2 show greater expression in PBMC from mild COVID-19 patients

**Fig. 2:**
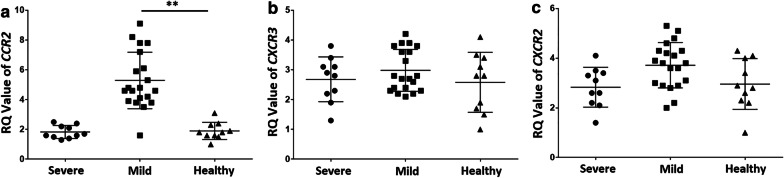
CCR2 show greater expression in PBMC from mild COVID-19 patients. RNA of PBMCs from severe COVID-19 patients (n = 10), mild COVID-19 patients (n = 20), and healthy controls (n = 10) was reverse transcribed into cDNA, and then RT-qPCR was performed to quantify chemokine receptor and GAPDH levels. **a** Expression levels of CCR2 is higher in PBMCs from mild COVID-19 patients. **b** CXCR2 and **c** CXCR3 levels did not differ between COVID-19 patients and healthy controls. **P < 0.01

Chemokines, when bound to their corresponding receptors, play a chemotactic role in immune cells. To further clarify the role of chemokines in mild COVID-19 disease, the expression levels of the receptors for MCP-1, IP-10, and IL-8, were assessed. Expression levels of the receptor of MCP-1, C-C motif receptor 2 (CCR2); the receptor for IP-10, chemokine (C-X-C motif) receptor 3 (CXCR3); and the receptor for IL-8, CXCR2 were assessed in the PBMCs from severe or mild COVID-19 patients compared to those from healthy controls. We observed that CCR2 expression levels (5.28 ± 1.89) were upregulated in mild COVID-19 patients compared to healthy controls (1.9 ± 0.57; P < 0.01), but there was no difference was observed between severe COVID-19 patients (1.83 ± 0.43) and healthy controls (Fig. 2a). Meanwhile, there was no difference in the expression levels of CXCR3 (Fig. 2b) and CXCR2 from PBMCs in either severe, or mild COVID-19 patients or in healthy controls (Fig. 2c).

### Higher expression of MCP-1 in mild COVID-19 patients was negatively correlated with IRF3 expression

**Fig. 3:**
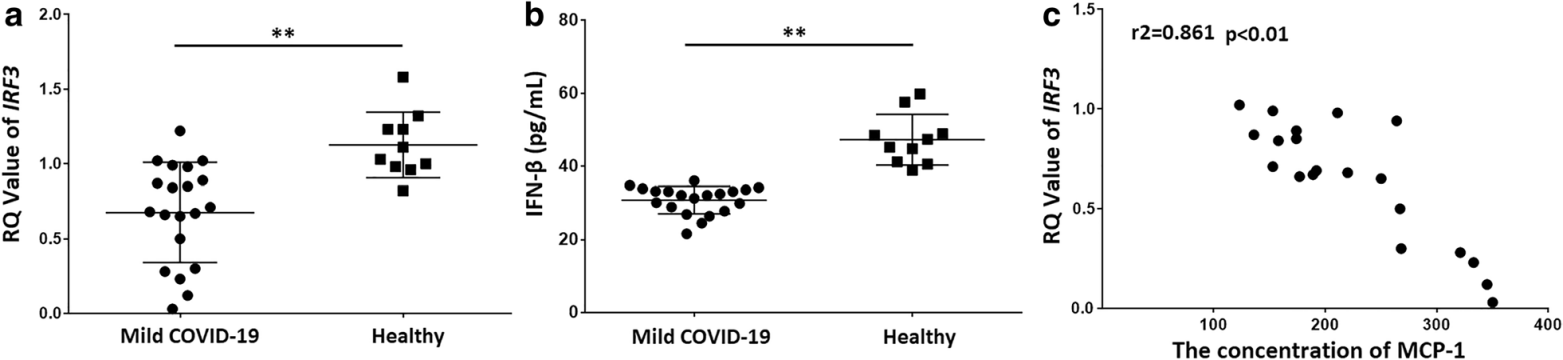
Higher expression of MCP-1 in mild COVID-19 patients was negatively correlated with IRF3 expression. **a** RNA samples were extracted from PBMCs of mild COVID-19 patients (n = 20) and healthy controls (n = 10), and *IRF3* was detected by RT-qPCR. *IRF3* expression was significantly downregulated in mild COVID-19 patients. **b** Cytokine levels in serum of study subjects were detected by ELISA. IFN-β levels in serum of peripheral blood from mild COVID-19 patients were lower than in peripheral-blood serum from healthy controls. **c** SCC analysis was performed to analyze serum MCP-1 and expression level of *IRF3*. *IRF3* expression was significantly negatively correlated with MCP-1 concentration. The correlation coefficient *r*^2^ represents the degree of correlation. **P < 0.01, *r*^2^ = 0.861

Transcriptional activation of IRFs results in the launch of general antiviral programs. We next explored the expression level of *IRF3*, an important gene in the interferon signaling pathway, in mild COVID-19 patients. Expression of *IRF3* was downregulated (0.67 ± 0.35 in mild COVID-19 patients vs 1.12 ± 0.21 in healthy controls; Fig. 3a; *P* < 0.01). Meanwhile, the downregulation of IFN-β was observed in mild COVID-19 patients (31.6 ± 3.7 in mild COVID-19 patients vs 47.3 ± 6.9 in healthy controls; Fig. 3b; *P* < 0.01). To clarify the relationship between serum MCP-1 and expression levels of *IRF3*, we performed SCC analysis using SPSS software. The results showed that *IRF3* downregulation was significantly negatively correlated with the expression levels of MCP-1 (Fig. 3c; *P* < 0.01, r^2^ = 0.861). Taken together, these results suggested that MCP-1 might play a role in the pathogenesis of mild COVID-19 disease and that higher expression thereof in these mild patients might be correlated with inhibition of IFN signaling.

## Discussion

Previous studies have shown that elevated levels of pro-inflammatory cytokines, such as IFN-γ, tumor necrosis factor alpha (TNF-α), IL-6 and IL-8, are associated with severe lung injury and adverse outcomes of SARS-CoV or MERS-CoV infection [18–20]. It has also been demonstrated that severe COVID-19 patients have higher concentrations of chemokines in their serum than do mild cases, suggesting that the magnitude of cytokine storm is associated with disease severity [6, 7].

Most cases of COVID-19 in Shiyan city are mild [4]. To further evaluate levels of chemokines in these patients, we detected levels of the chemokines MCP-1, IP-10, and IL-8 in the serum of mild COVID-19 patients admitted to Xiyuan Hospital and Renmin Hospital in Shiyan City. MCP-1, belongs to the C-C chemokine family and is a powerful monocyte chemotactic factor that is constitutively produced or induced by oxidative stress, cytokines, or growth factors. Monocytes and macrophages are the main source of MCP-1, which regulates the migration and infiltration of monocytes, memory T cells, and natural-killer (NK) cells [21]. Huang et al. found that MCP-1 expression levels were higher in patients with COVID-19 and even higher among those admitted to the ICU [10]. It has been reported that MCP-1 expression increases rapidly in the early acute phase of infection and then progressively decreases as the disease advances [22]. Xiong et al. detected elevated levels of MCP-1 in bronchoalveolar lavage fluid from COVID-19 patients and found it to be associated with the pathogenesis of the SARS-CoV-2 [23]. Elevated levels of MCP-1 have also been detected in the lung tissue of patients infected with virus [24]. Therefore, monitoring MCP-1 levels early and acting upon any elevation thereof might be a viable strategy to prevent COVID-19 from progressing from mild to severe. IP-10 was initially identified as a chemokine whose secretion is induced by IFN-γ; it is secreted by neutrophils, endothelial cells, keratinocytes, fibroblasts, dendritic cells, astrocytes, and hepatocytes. Through its binding to CXCR3, it regulates immune system responses by activating and recruiting leukocytes, including T cells, monocytes, and NK cells [25]. Therefore, IP-10 and CXCR3 play key roles in recruiting leukocytes to inflamed tissues and in perpetuating inflammation, thereby making a major contribution to tissue damage [25]. Further comparison between ICU and non-ICU COVID-19 patients showed that plasma concentrations of IP-10 were higher in the former [10], suggesting a relationship between IP-10 and COVID-19 severity. Liu et al. associated elevated serum IP-10 levels with higher viral load and greater lung damage in patients with SARS-CoV-2 [26]. Recent reports suggest that expression of IL-8 is also higher in patients with severe COVID-19 [10]. Our results proved that MCP-1, IP-10 and IL-8 were upregulated in such patients (Fig. 1). It has been suggested that chemokines play an important role in patients with severe COVID-19. In our study, MCP-1 was more highly expressed in patients with mild cases of COVID-19 (Fig. 1a), while IP-10 and IL-8 were upregulated in severe COVID-19 patients, but not in mild ones (Fig. 1b, c). Next, we measured expression levels of the receptors for MCP-1, IP-10, and IL-8 [27], respectively. We observed upregulation of CCR2 in mild COVID-19 patients compared to healthy controls, but saw no difference between severe patients and healthy controls (Fig. 2). This demonstrated that MCP-1 may participate in the pathogenesis of mild COVID-19 diseases.

Engagement of virus-specific RNA structures culminates in oligomerization of these receptors and activation of downstream transcription factors, most notably IRFs and NF-κB. We found that in mild COVID-19 patients with higher levels of MCP-1, expression of *IRF3*, an important gene in the interferon signaling pathway, was downregulated (Fig. 3a). There was no significant difference in expression of NF-κB between these patients and healthy controls (data not shown). IFN-β levels were lower in serum of peripheral blood from mild COVID-19 patients than in that from healthy controls (Fig. 3b). Meanwhile, *IRF3* downregulation was significantly negatively correlated with MCP-1 level (Fig. 3c). We also evaluated the expression levels of *IRF3* in severe COVID-19 patients, but found no relationship between MCP-1 and the expression of *IRF3* (data not shown). Therefore, we considered that the higher expression of MCP-1 in mild COVID-19 patients might be correlated with the inhibition of IFN signaling. Michael J, et al [28] demonstrated that IFN-β can induce MCP-1 transcription in bone marrow derived macrophages (BMDMs). Our results demonstrated a negative correlation between MCP-1 level in peripheral blood and *IRF3* expression level in PBMCs from mild COVID-19 patients in Shiyan City. Therefore, we suggest that IFN might be used to treat mild COVID-19 patients following detection of upregulation of MCP-1 and downregulation of *IRF3*.

Other studies have already established that SARS-CoV-2 has greater sensitivity to type I IFN than SARS-CoV does [24]. Pretreatment with IFN-α or IFN-β drastically reduces viral titers. These findings also suggest that type I IFN could be effective as a prophylactic agent or an early treatment option for SARS-CoV-2. However, delayed IFN administration was of no benefit over a placebo [29]. Channappavanar et al. showed that delayed type I IFN expression can be detrimental in mice in the context of SARS-CoV-1 infection [30]. The timing of interferon exposition could be critical for controlling the virus and avoiding immunopathogenesis.

## Conclusion

In summary, we found that higher expression of MCP-1 in mild COVID-19 patients might be correlated with inhibition of IFN signaling.

## Data Availability

All data generated or analyzed during this study are included in this published article.

## Abbreviations

SARS-CoV-2: Severe acute respiratory syndrome coronavirus 2
MERS-CoV: Middle East respiratory syndrome coronavirus
ICU: Intensive-care unit
WHO: World Health Organization
*IRF3*/7: Interferon regulatory factor 3/7
PBMCs: Peripheral-blood mononuclear cells
ELISA: Enzyme-linked immunosorbent assay
MCP-1: Monocyte chemotactic protein 1
IP-10: Interferon-inducible protein-10
IL-8: Interleukin-8
CCR2: C-C motif receptor 2
CXCR: Chemokine (C-X-C motif) receptor
BMDMs: Bone marrow–derived macrophages
